# Interaction Between Mesenchymal Stem Cells and Retinal Degenerative Microenvironment

**DOI:** 10.3389/fnins.2020.617377

**Published:** 2021-01-21

**Authors:** Yu Lin, Xiang Ren, Yongjiang Chen, Danian Chen

**Affiliations:** ^1^The Research Laboratory of Ophthalmology and Vision Sciences, State Key Laboratory of Biotherapy, West China Hospital, Sichuan University, Chengdu, China; ^2^The Department of Ophthalmology, West China Hospital, Sichuan University, Chengdu, China; ^3^The School of Optometry and Vision Science, University of Waterloo, Waterloo, ON, Canada

**Keywords:** mesenchymal stem cells, retinal degenerative diseases, interaction, trophic, inflammation, tunneling nanotubes, immunomodulation, licensing

## Abstract

Retinal degenerative diseases (RDDs) are a group of diseases contributing to irreversible vision loss with yet limited therapies. Stem cell-based therapy is a promising novel therapeutic approach in RDD treatment. Mesenchymal stromal/stem cells (MSCs) have emerged as a leading cell source due to their neurotrophic and immunomodulatory capabilities, limited ethical concerns, and low risk of tumor formation. Several pre-clinical studies have shown that MSCs have the potential to delay retinal degeneration, and recent clinical trials have demonstrated promising safety profiles for the application of MSCs in retinal disease. However, some of the clinical-stage MSC therapies have been unable to meet primary efficacy end points, and severe side effects were reported in some retinal “stem cell” clinics. In this review, we provide an update of the interaction between MSCs and the RDD microenvironment and discuss how to balance the therapeutic potential and safety concerns of MSCs' ocular application.

## Introduction

Retinal degenerative diseases (RDDs), including age-related macular degeneration (AMD), retinitis pigmentosa (RP), Stargardt disease (STGD), and Leber congenital amaurosis (LCA), are some of the leading causes of irreversible vision loss worldwide (Veleri et al., 2015). In addition, diabetic retinopathy (DR), glaucoma, and some other retinopathies can damage the retinal neurons and are thus also considered RDDs (Gorbatyuk and Gorbatyuk, 2013; Mead et al., 2015). These diseases primarily damage the ganglion cells (RGCs), photoreceptors (rods and cones), or retinal pigment epithelium (RPE) cells (Mead et al., 2015) but can also induce some secondary cellular reactions such as neuro-inflammation, microglial activation, and retinal gliosis (activation of the Müller glial cells) (Ghosh et al., 2018; Rashid et al., 2018).

While there are currently no effective treatments for RDDs as the human retina has no regenerative ability, new approaches—such as gene therapy, neuroprotection, anti-VEGF (vascular endothelium growth factors), and stem cell therapy—have the potential to delay vision loss or even to restore vision (Dalkara et al., 2016). Gene therapy can cure certain types of RDDs caused by single recessive gene defects, if applied in the early stages of the disease process while the photoreceptors are still alive (Scholl et al., 2016). Neuroprotective reagents are believed to be the best method to treat progressive photoreceptor cell degradation; however, they are unable to regenerate previously lost retinal neurons (Oswald and Baranov, 2018). Anti-VEGF therapy is the standard of care for neovascular AMD; it is also used to treat DR and many other retinal neovascular conditions. However, it has no effect on dry AMD and non-proliferative DR (Ferrara and Adamis, 2016).

In contrast to these treatments, cell-based therapy presents an opportunity for preserving or restoring vision in advanced stages of retinal degeneration and can be utilized even when photoreceptor cell degeneration has already occurred (Scholl et al., 2016). There are numerous pre-clinical and clinical trials regarding the application of several types of cells—including neural stem cells, mesenchymal stromal/stem cells, embryonic stem cells, and induced pluripotent stem cells—in RDDs (Li et al., 2016b; Ding et al., 2017; Enzmann et al., 2017; Mclelland et al., 2018; Jin et al., 2019; Shen, 2020). Stem cells have the capacity for self-renewal and are able to differentiate into multiple cell types (Ludwig et al., 2019). Degenerated retinal cells can benefit from stem cell transplantation, which can act as vehicles for drug delivery, immune modulators, or sources for direct tissue regeneration (Aharony et al., 2017).

Mesenchymal stem or stromal cells (MSCs) are multipotent cells isolated from a variety of tissues, such as adult bone marrow (bone marrow-derived MSC, BM-MSC), adipose tissue (adipose-mesenchymal derived stem cells, ASC), and dental pulp (dental pulp stem cells, DPSC) (Mead et al., 2013, 2017). They can also be isolated from neonatal tissues and fluids, such as umbilical cord (umbilical cord-derived MSCs, UC-MSC), Wharton's jelly MSCs (WJ-MSCs), amniotic membrane, amniotic fluid, and placenta (Ding et al., 2011). Although MSCs are derived from different tissues, they share some common features. In 2006, the International Society for Cellular Therapy established the minimal criteria for designating a cell as MSC (Dominici et al., 2006). MSCs are able to adhere to plastic in standard culture conditions and have the potential to differentiate into adipocytes, chondroblasts, and osteoblasts. In general, they do not express hematopoietic and endothelial cell markers, such as CD34 CD45, CD11b, CD11c, CD14, CD19, CD79α, CD86, and HLA class II molecules, but they do phenotypically express a distinct set of cell surface markers for CD105, CD90, CD44, CD9, and CD73 (Dominici et al., 2006; Marino et al., 2019; Naji et al., 2019). The low immunogenicity (low levels of HLA class I and absence of HLA class II expression) makes MSCs a good candidate for cell transplantation (Ryan et al., 2005).

In addition to their wide distribution and ease to harvest, MSCs are also known to possess minimal susceptibility to malignant transformation and are capable of avoiding immune rejection (Ding et al., 2017). MSCs have therapeutic efficacy to promote regeneration of multiple tissues and cells, including central nervous system (CNS) neurons (Nakano et al., 2016). Thus, allogeneic or autologous cell transplantation of MSCs shows promises for potential therapeutic applications in RDDs. Indeed, several pre-clinical trials of MSCs in the treatment of rodent RDDs (such as streptozotocin or STZ-induced diabetic rodent models, rodent retinal degeneration models, and rodent glaucoma and retinal ischemia models) generated encouraging results (Lund et al., 2007; Guan et al., 2013; Tzameret et al., 2014; Ezquer et al., 2016; Mead et al., 2016; Roth et al., 2016). More than 40% of clinical trials of stem cell therapy for retinal diseases are also using bone marrow or umbilical cord-derived stem cells (Park et al., 2017; Shen, 2020).

However, there are several reports that RDD patients lost their vision after receiving intraocular injection of autologous bone marrow/adipose tissue–derived stem cells (Leung et al., 2016; Kuriyan et al., 2017; Rong et al., 2018; Khine et al., 2020). These devastating outcomes are primarily due to rhegmatogenous retinal detachments with severe proliferative vitreoretinopathy (PVR). The subretinal delivery of human umbilical tissue-derived cells (palucorcel) into eyes with geographic atrophy of AMD patients was also associated with a high rate of retinal perforation and detachment (Ho et al., 2017). From a scientific perspective, a probable cause is the trans-differentiation of injected MSCs into myofibroblast-like cells, which can induce fibrosis or PVR. Another possible mechanism is the toxicity to the retina or optic nerve caused by the injected material, which may have included enzymes used in the preparation process (such as trypsin). Periocular injection of autologous bone marrow stem cells to treat RP has also been reported to induce central retinal artery occlusion and vision loss (Boudreault et al., 2016). These severe complications call for a better understanding of the interaction between the donor MSCs and the degenerative host retinal environment. It is also imperative to have stringent quality control of MSC preparation and careful surgical procedures for ocular or intraocular delivery of MSCs (Apatoff et al., 2018; Singh et al., 2020). The aim of this brief review is to provide an update on the interaction between MSCs and the RDD microenvironment and to discuss methods by which to balance the therapeutic potential and safety concerns of MSCs ocular application.

## The Interaction Between MSCs and the Retinal Environment

**Figure 1:**
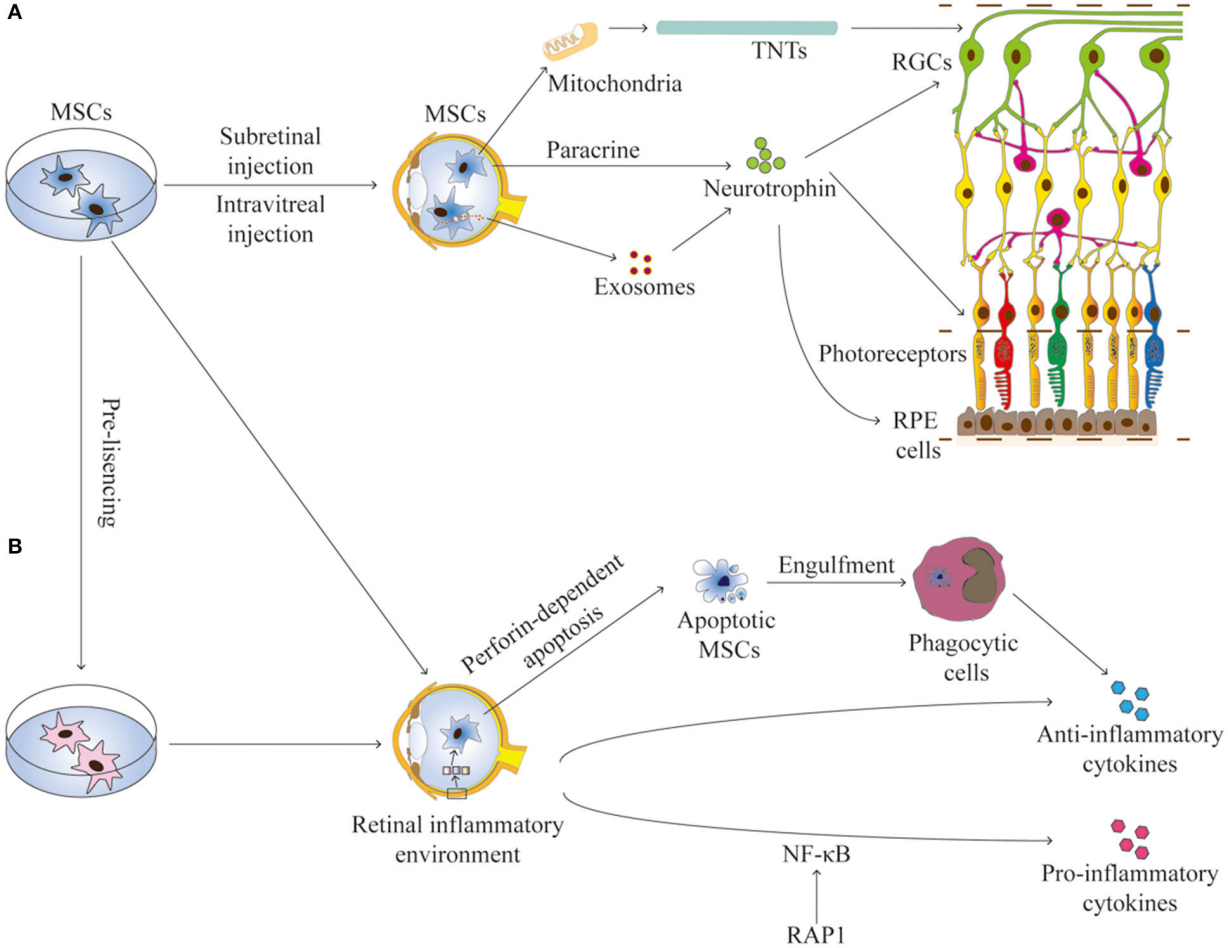
The interactions between MSCs and the retinal environment. **(A)** MSCs can secrete neurotrophins, produce exosomes, or transfer mitochondria by TNTs, all contributing to the trophic effects on RGCs, photoreceptors, and RPE cells in RDDs. **(B)** MSCs have immunomodulation properties to produce anti-inflammation or pro-inflammation cytokines. Apoptotic MSCS can induce immunosuppressive effects through phagocytic cells. The secretory activity of MSCs is strongly influenced by the retinal environment. Pre-licensing or selective suppression Rap1 may enhance the immunosuppressive effects of MSCs. TNTs, tunneling nanotubes; RGCs, retinal ganglion cells; RPE, retinal pigment endothelium.

The communication between MSCs and host cells is mainly mediated by paracrine activity, tunneling nanotubes (TNTs), and extracellular vesicles (such as exosomes and microvesicles) that contain reparative molecules (Spees et al., 2016; Holan et al., 2019). The secretory activity of MSCs is strongly influenced by the retinal environment (Holan et al., 2019). Collectively, these mechanisms confer MSCs both trophic and immunomodulatory properties, which are essential for their application for RDDs (Figure 1).

### MSCs Promote Retinal Cell Survival or RPE Phagocytic Function

MSCs can protect retinal cells by secreting growth factors, which can be classified into two categories (Murray et al., 2014). In the first category are factors promoting cell proliferation, including transforming growth factor-alpha (TGF-α), TGF-β, hepatocyte growth factor (HGF), epidermal growth factor (EGF), and fibroblast growth factor-4 (FGF-4); the second category consists of factors enhancing angiogenesis, including VEGF, interleukin-8 (IL-8), and insulin-like growth factor-1 (IGF-1) (Murphy et al., 2013; Kocan et al., 2017). Under stress conditions, such as hypoxia and growth factor deprivation, MSCs can increase the secretion of these factors (Anderson et al., 2016). The trophic effects of MSCs on retinal ganglion cells (RGCs), photoreceptors, RPE cells, and multiple cell types in diabetic retinopathy have been observed in several pre-clinical studies (Figure 1A).

#### A-Retinal Ganglion Cells

Intravitreal injection of BM-MSCs promoted the survival of RGCs for at least four weeks by secretion of neurotrophic factors in an ischemia and reperfusion rat model (Li et al., 2009) and increased the overall RGC axon survival and reduced the RGC axon loss rate in a laser-induced ocular hypertensive glaucoma rat model (Johnson et al., 2010). However, intravenously injected MSCs did not migrate to the injured eyes and had no effects on optic nerve damages (Johnson et al., 2010). Intravitreal transplants of DPSCs promoted significant RGC survival and axon regeneration in a surgically induced optic nerve crush injury rat model, where the effects were mediated by neurotrophins and abolished after TrK receptor blockade (Mead et al., 2013). These results were also verified by a coculture study of the porcine retinal cells and human MSCs, separated in a Transwell system, which suggested that MSCs produce brain-derived neurotrophic factor (BDNF) and ciliary neurotrophic factor (CNTF) (Labrador-Velandia et al., 2019).

Interestingly, overexpression of neurotrophin in MSCs can enhance these protective effects. Intravitreal transplanted MSCs engineered to express BDNF and green fluorescent protein (GFP) survived in rat eyes with chronic hypertension and protected retinal and optic nerve function and structure (Harper et al., 2011). Similarly, intravitreally injected MSCs engineered to overexpress nerve growth factor (NGF) survived and promoted RGC survival and regeneration in a NGF-dependent manner (Levkovitch-Verbin et al., 2010).

There is evidence that exosomes mediate the effect of MSCs on damaged RGCs (Mathew et al., 2019). Exosomes are membrane-enclosed extracellular vesicles that contain mRNA, microRNA, and proteins including neurotrophins. MSC-derived exosomes can rapidly reach RGCs to supply them with neurotrophins and suppress their cell death (Mead and Tomarev, 2017). TNT-mediated mitochondria transfer from MSCs to RGCs is another mechanism to explain the protective effects. TNTs are communicating intercellular transport networks formed between many cells (Rustom et al., 2004). The TNTs are tubular structures, with diameters varying between 50 and 1,500 μm, which can span hundreds of microns between two cells. These structures allow immediate intercellular communication. Moreover, the fibers formed from the cytoskeleton allow the transfer of cytosolic material. Mitochondria can be transferred from MSCs to injured target cells to restore their function (Spees et al., 2006; Jiang et al., 2016; Vignais et al., 2017). In an inflammation-related RGC degeneration mouse model (NADH dehydrogenase ubiquinone Fe-S protein 4, or Ndufs4 knockout mice), intravitreally injected human iPSC-MSCs could transfer functional mitochondria across the inner limiting membrane to RGCs and restore the RGC function against mitochondrial dysfunction-induced inflammation (Jiang et al., 2019). While the results from these pre-clinical studies support the usage of MSCs in the treatment of glaucoma, there are currently no reports regarding their usage in clinical trials (Harrell et al., 2019).

#### B-Photoreceptors

Subretinal transplanted mouse GFP-labeled BM-MSCs into RP model Rho^−/−^ mice integrated into the retinal pigment epithelium layer and protected the photoreceptor from death (Arnhold et al., 2007). Similarly, subretinal transplantation (trans-scleral and trans-choroidal approach) of mouse BM-MSCs into the Royal College of Surgeons (RCS) rat delayed retinal degeneration and preserved retinal function (Inoue et al., 2007). It has been demonstrated *in vitro* that the conditioned medium of the MSCs delays photoreceptor cell apoptosis, suggesting that secreted factor(s) from MSCs promote photoreceptor cell survival (Inoue et al., 2007). Subretinal or intravitreally injected human BM-MSCs into RCS rat can delay photoreceptor death for about 12–20 weeks (Tzameret et al., 2014). Subretinal transplantation of rat MSCs or engineered erythropoietin (EPO)-expression rat MSCs into a sodium iodate (SI)-induced rat model of retinal degeneration protected RPE and retinal neurons; EPO expression MSCs had an even greater effect (Guan et al., 2013). Subretinal transplantation of human adipose derived stem cell (hADSCs) (Li et al., 2016a) and human periodontal ligament-derived stem cells (hPDLSCs) (Huang et al., 2017) also protected the photoreceptors in RCS rats. It has been suggested that hADSCs can suppress the expressions of Bax, Bak, and Caspase 3 and produce VEGF, HGF, and pigment epithelium-derived factor (PEDF), all of which may contribute to their neuroprotective effects (Li et al., 2016a).

Interestingly, other stem cells derived from bone marrow (not MSCs) can also protect photoreceptors. Intravitreally injected autologous bone marrow–derived lineage-negative hematopoietic stem cells prevented cone loss in two murine models of retinitis pigmentosa (rd1 and rd10) (Otani et al., 2004). Bone marrow–derived endothelial progenitor cells (EPC) with low aldehyde dehydrogenase (Aldh) activity, when injected intravitreally into rd1 mice, protected the retinal vasculature and photoreceptors (Fukuda et al., 2013).

#### C-RPE Cell Function

Subretinal injection of human umbilical tissue-derived cells (hUTCs) in the RCS rat model of retinal degeneration can preserve photoreceptors and visual function (Lund et al., 2007), as hUTCs can rescue the phagocytic dysfunction in RCS RPE cells *in vitro* by secreting several trophic factors—including BDNF, HGF, and GDNF—as well as opsonizing bridge molecules MFG-E8, Gas6, TSP-1, and TSP-2 (Cao et al., 2016). These trophic factors—derived from the conditioned medium of hUTCs—are also beneficial to the phagocytic function of human RPE cells isolated from the post-mortem eyes of AMD-affected subjects (Inana et al., 2018). In a phase 2b clinic trial, while hUTCs (palucorcel) were delivered successfully to the targeted subretinal space for most participants, improvements in GA (geographic atrophy of AMD) area or visual acuity were not demonstrated; thus, no apparent therapeutic effect was observed (Heier et al., 2020).

#### D-Multiple Cell Types in Diabetic Retinopathy

Intravitreal injection of human ASCs or cytokine-primed ASCs conditioned media (ASC-CM) into STZ-induced diabetic athymic nude rats (Rajashekhar et al., 2014) and diabetic Ins2^Akita^ mice (Elshaer et al., 2018), improved ERG b-wave amplitudes and vascular leakage, and reduced apoptotic cells around the retinal vessels. ASC-CM (but not ASCs itself) can improve retinal gliosis, DR-related gene expression profile, and mouse visual acuity. ASC-CM had high levels of anti-inflammatory proteins, including indoleamine 2, 3-dioxygenase 1 (IDO-1), IDO-2, and TSG-6 (Elshaer et al., 2018). Intravitreally injected ASCs also reduced oxidative damage and increased the intraocular levels of several potent neurotrophic factors—including NGF, bFGF, and GDNF—in a diabetic mouse model, thus preventing RGC loss (Ezquer et al., 2016). Interestingly, intravitreally injected BM-MSCs were found to integrate into the inner retina, differentiate into retinal glial cells, and improve ERG amplitude, thereby protecting vision in a STZ-induced mouse model (Çerman et al., 2016). Excitingly, intravenously administrated autologous BM-MSCs were found to be beneficial in non-proliferative DR (NPDR) patients, showing significant improvements in macular thickness and best-corrected visual acuity (BCVA) from baseline (Gu et al., 2018).

### MSCs Regulate Retinal Inflammation and Immune Responses

When exposed to an inflammatory environment, MSCs can modulate local and systemic, innate, and adaptive immune responses through the release of various mediators, which include cytokines, chemokines, and some metabolites, such as IDO, IL-6, PGE2, and TGF-β1. While immunosuppression is mainly mediated by IDO in human MSCs, it is mediated by inducible nitric oxide synthase (iNOS) in mouse MSCs (Ren et al., 2009). Interestingly, apoptotic MSCs also have some immunosuppressive functions *in vivo*. MSCs can be actively induced to undergo perforin-dependent apoptosis by recipient cytotoxic cells; apoptotic MSCs are engulfed by host phagocytic cells that, by producing IDO, became the ultimate effectors of immunosuppression (Galleu et al., 2017). Thus, host phagocytes are crucial for generating an immunosuppressive environment by clearing apoptotic MSCs (De Witte et al., 2018). MSCs can suppress the proliferation of T cells, B cells, and natural killer cells, inhibit the differentiation and maturation of monocyte-derived dendritic cells, and promote the generation of regulatory T cells (Melief et al., 2013). TNT-mediated mitochondria transfer also plays a role in immunomodulation. Mitochondrial transfer from MSCs to CD4^+^ T cells increases the expression of factors involved in T-cell activation and differentiation, including FoxP3, IL2Ra, CTLA4, and TGF-β1, leading to an increase in the suppressive regulatory T cell population (Court et al., 2020). Both pro- and anti-inflammatory effects can be observed in the application of MSCs to the retina (Figure 1B).

#### A-MSCs Suppress Retinal Inflammation

Inflammation is a major mechanism in the pathogenesis of RDDs, including DR, AMD, and RP (Akhtar-Schafer et al., 2018; Arroba et al., 2018). The upregulated pro-inflammatory mediators (such as cytokines) induce breakdown of the blood–retinal barrier (BRB) and immune cell infiltration (such as macrophages and microglia), which form an inflammatory retinal microenvironment (Kauppinen et al., 2016; Mesquida et al., 2019). MSCs can regulate this inflammatory retinal environment through their immunomodulation properties (Shi et al., 2018). For instance, intravitreal injection of pro-inflammatory cytokines (IL-1β, TNF-α, and IFN-γ) into mice induces retinal edema, vessel dilatation, and microglial accumulation in the retina, resulting in a mouse model of retinal inflammation (Mugisho et al., 2018). Intravitreal injection of BM-MSCs into this mouse model reduced the retinal expression of pro-inflammatory molecules such as IL-1α, IL-6, iNOS, TNF-α, and VEGF and reduced macrophage infiltration (Hermankova et al., 2019). MSC-derived exosomes can also suppress laser injury-induced mouse retinal inflammation; MCP-1 is likely the major cytokine mediating this effect (Yu et al., 2016). TNT-mediated mitochondria transfer also plays a role in the immunomodulatory effect in the Ndufs4 knockout mouse model to reduce inflammation and RGC degeneration (Jiang et al., 2019).

#### B-MSCs Promote Retinal Inflammation

The immunomodulatory capabilities of MSCs are not constitutive but rather are licensed by inflammatory cytokines (Boland et al., 2018); the effects of MSCs may vary depending on the local inflammatory environment within the targeted tissues (Han et al., 2014; Shi et al., 2018). Indeed, intraocular injection of MSCs generally induces retinal inflammation in wild-type animals. For instance, Wharton's jelly MSCs (hWJMSCs), injected intravitreally into a rat model of optic nerve axotomy-induced RGC degeneration, secrete anti-inflammatory molecules and trophic factors, but in naive retinas, they instead induce a massive migration of microglial/macrophages from the choroid to the inner retina, disrupting the retinal architecture—a typical retinal inflammatory response (Millán-Rivero et al., 2018). Similarly, intravitreally injected human BM-MSCs induce tractional epiretinal membrane formation in Nod-SCID mice (Park et al., 2017) and induce gliosis-mediated retinal folding, upregulation of intermediate filaments, and recruitment of macrophages in C57BL/6 mice (Tassoni et al., 2015). Retinal glial activation and elevation of IL-1β, C3, arginase 1, and heat shock protein 90 were also detected in SD rats with intravitreally injected BM-MSCs (Huang et al., 2019).

#### C-NF-κB/Rap1 Inhibition and Pre-licensing

The method by which to simultaneously enhance the anti-inflammatory properties and inhibit the pro-inflammatory capabilities of MSCs is a pertinent issue to be addressed for the therapeutic use of MSCs in the retina. There are two possible strategies (Figure 1B). The first one is based on the NF-κB signaling pathway, a pivotal mediator of inflammatory responses (Liu et al., 2017). NF-κB mediates cytokine/growth factor secretion by MSCs (Mutt et al., 2012); thus, inhibition of NF-κB pathways may suppress the pro-inflammatory capability of MSCs. However, as this pathway is crucial in maintaining normal host defense and generating innate immune responses, complete blockade of NF-κB activity is not feasible (Poon et al., 2015; Liu et al., 2018). Rap1, a member of the telomeric shelterin complex, is a novel modulator involved in the NF-κB pathway (Teo et al., 2010). Selective inhibition of Rap1 in BM-MSCs can decrease NF-κB sensitivity to pro-inflammatory cytokines and enhance MSC-based therapeutic efficacy for myocardial infarction (Zhang et al., 2015). It would be interesting to test if Rap1 suppression can improve the effects of MSCs application in the retina.

Another strategy is based on the fact that the inflammatory environment can actually enhance the immunosuppression function of MSCs (Naji et al., 2019). MSCs can be polarized to pro- or anti-inflammatory phenotypes by pre-conditioning with cytokines, including IFN-γ, TNF-α, or IL-17 (Hemeda et al., 2010), and by signaling through Toll-like receptors (Mastri et al., 2012). Similarly, pre-stimulation of MSCs with anti-inflammatory factors such as TGF-β reversed the immunosuppressive effect of MSCs and conferred a pro-inflammatory phenotype (Xu et al., 2014). It has been suggested that *in vitro* pre-stimulation of MSCs with appropriate pro-inflammatory factors (pre-licensing) may obtain optimal therapeutic effects *in vivo* (Boland et al., 2018; Naji et al., 2019). IFN-γ is the most commonly used cytokine for pre-licensing or priming. While IFN-γ pre-licensing enhances IDO expression of cryopreserved human MSCs *in vitro*, surprisingly these pre-licensed MSCs lose effectiveness *in vivo*; they rescued fewer RGCs than either fresh or unlicensed cryopreserved MSCs in a mouse model of retinal ischemia/reperfusion injury (Burand et al., 2017). While MSCs licensed with IFN-γ are known to increase expression of immunosuppressive factors, the expression of MHC-I and MHC-II molecules was also enhanced in the surface of these cells, which may induce strong immune rejection (Ankrum et al., 2014a). Further investigations are needed to optimize the pre-licensing procedures (Boyt et al., 2020).

#### D-Immune Rejection and MSC Transplantation

As most clinical applications of MSCs in the retina are allogeneic transplantations, immune rejection is an issue that needs to be considered. The vitreous cavity and subretinal space are advantageous for stem cell transplantation as they are immune privileged (Mead et al., 2015). The low immunogenicity and immunosuppressive property of MSCs also reduce the chance of immune rejection (Li et al., 2015).

Indeed, several pre-clinical studies injecting human MSCs into the eyes of rodent disease models (xenotransplantation) without using immunosuppressant have not observed obvious rejection (Lund et al., 2007; Tzameret et al., 2014; Li et al., 2016a; Elshaer et al., 2018). Subretinally administered human adult bone marrow-derived somatic cells (hABM-SCs) can achieve similar therapeutic benefits to protect the rods with or without cyclosporine A in the RCS rats (Lu et al., 2010). Interestingly, these effects do not require the cells to survive for a long period of time (Lu et al., 2010). This observed therapeutic benefit in the absence of long-term survival of transplanted cells is consistent with the so-called hit-and-run mechanism, mediated by the production of exosomes or secretion of trophic and immunomodulatory factors during the initial days following MSC injection (Von Bahr et al., 2012; Ankrum et al., 2014b) and may also be related to the immunomodulation effects mediated by apoptotic MSCs (Galleu et al., 2017).

However, immune privilege of the subretinal space and vitreous is determined by the retinal microenvironment and the integrity of BRB, which may be disrupted by RDDs or surgical procedures (Jiang et al., 1993). It has also been demonstrated that MSCs, like all somatic tissues, express MHC class I molecules constitutively and have the ability to express MHC class II when exposed to inflammatory cues such as INF-γ (Galipeau and Sensébé, 2018). Indeed, several studies used immunosuppressive drugs after subretinal transplantation of MSCs and showed that these treatments are efficacious in the prevention of immune rejection and increase the survival rates of MSCs, indicating that immune rejection does exist in these immune-privileged tissues (Francis et al., 2009; Xian and Huang, 2015). It is still controversial whether or not immunosuppression should be used after MSC transplantation; it may depend on the integrity of the RPE layer and BRB (Xian and Huang, 2015). If the RPE layer or BRB is intact, immune suppression is not necessary; if not, as in the RDDs, an immunosuppressant is needed (Oner et al., 2016). While it is not clear if extended MSC persistence or immune tolerance to MSCs will translate to a sustained therapeutic effect and improve clinical outcomes, conventional wisdom suggests that the beneficial effects of MSC therapy could be boosted by extending their persistence after injection (Ankrum et al., 2014b). In order to minimize cell rejection until the BRB is reestablished, many clinical trials of allogeneic MSC application for retinal diseases required systemic immunosuppressive therapy for the first 2–3 months after cell transplantation surgery (Oner et al., 2016; Park et al., 2017). However, the Palucorcel clinical trial (clinicaltrials.gov identifier NCT01226628) has no protocol-specified systemic immunosuppression included in the study, and no apparent effect of treatment was observed in the study (Ho et al., 2017; Heier et al., 2020).

## Challenges For MSC Therapies of RDDs

Early encouraging pre-clinical animal results in the therapeutic use of MSCs have prompted great interest in exploring their potential for promoting retinal cell survival and modulating retinal inflammation. There are currently many MSC clinical trials for retinal diseases around the world (Shen, 2020; Singh et al., 2020). While the safety profiles shown in clinical trials are generally promising, some outcomes of advanced clinical trials have fallen short of expectations (Heier et al., 2020). Overenthusiasm and optimism surrounding stem cell therapies have also caused a surge in for-profit “stem cell” clinics globally, forming a direct-to-consumer marketplace for autologous stem cell interventions (Turner, 2018; Nirwan et al., 2019; Hwang et al., 2020). These clinics are run without the oversight of regulatory agencies and offer scientifically unproven retinal cell therapy products, which are not prepared in a standard and rigorous manner (Shen, 2020). The surgical procedures involved in the delivery of MSCs to vitreous, subretinal space, or periocular space also have significant risks, such as retinal detachment, PVR, and retinal perforations (Singh et al., 2020). Overall, there are still challenges with regard to the heterogeneity in the MSC product and the routes of delivery for MSCs (Galipeau and Sensébé, 2018; Shen, 2020; Singh et al., 2020).

### Overcoming the Heterogeneity in the MSC Product

MSCs are an inherently heterogeneous population of cells whose therapeutic potency varies with the characteristics of the donor, tissue of origin, isolation method, and *in vitro* preparation methods (e.g., cell culture protocol and scale-up). The common sources of MSCs for clinical trials of retinal degeneration therapy are bone marrow (BM) stem cells (Cotrim et al., 2017; Park et al., 2017), adipose-derived MSCs (ASC) (Oner et al., 2016, 2018) and umbilical cord-derived MSCs (such as UC-MSC or palucorcel) (Heier et al., 2020). They differ in the ease and efficiency of harvesting, proliferative ability and senescence, and paracrine activities, which greatly affect their therapeutic potency (Fričová et al., 2020). For example, BM aspirate is difficult to harvest and contains only 0.001–0.01% MSCs in the overall cell population; an intensive culturing process to expand the MSCs is required for clinical use. ASCs can be harvested easily from a small area under local anesthesia—an ASC harvest obtains a 500 times greater yield of MSCs than an equivalent amount of BM aspirate; thus, ASCs are a better source for autologous clinical use. UC-MSC harvesting is the least invasive and can produce a large amount of MSCs which can minimize the need to extensively expand the cells for allogenic use. In general, UC-MSCs proliferate faster than ASCs, and ASCs proliferate faster than BM-MSCs. UC-MSCs, ASCs, and BM-MSCs have been found to have senescence landmarks starting at passage 16, 8, and 7, respectively. Senescence can influence the therapeutic effectiveness, number, and maximum lifespan of the MSCs. ASCs have a relatively low secretion rate of pro-angiogenic molecules and cytokines, and thus they might be less suited to suppressing inflammation. UC-MSCs can secrete more angiogenic, neuroprotective factors, which make them an attractive option in RDD therapy. The potential concern for UC-MSC transplantation is that, as a type of non-neuronal or non-terminally differentiated cell, they may retain a capacity for continued proliferation inside the eye after transplantation (Fričová et al., 2020; Singh et al., 2020).

The obvious heterogeneity of the MSC product introduced during the manufacturing process emphasizes the need for characterizing and controlling the therapeutic potency of MSCs. Quality control protocols to standardize MSC product potency can reduce the risk of clinical failure (Trivedi et al., 2019). The most widely used potency assay for the MSC product is based on *in vitro* inhibition of T cell proliferation using activated CD4^+^ T cells (Bloom et al., 2015). While potency assays may improve product quality by excluding MSCs with low potential therapeutic efficacy, alternative strategies are needed to generate high-quality MSCs in sufficient quantities for large clinical trials (Levy et al., 2020).

In order to overcome MSC product heterogeneity and generate homogeneous, standardized high-quality MSCs, human-induced pluripotent stem cell (iPSC)-derived MSCs have been proposed as an unlimited source of cells for therapeutic applications in regenerative medicine. These cells possess better cell quality with batch-to-batch consistency and higher proliferative potential and display stronger immunomodulation effects (Zhang et al., 2012; Sabapathy and Kumar, 2016). Cymerus™ iPSC-MSCs significantly prolong survival in a pre-clinical humanized mouse model of graft-vs-host disease (GVHD) (Ozay et al., 2019). Good manufacturing practice (GMP)-grade iPSC-MSCs have already been used in clinical trials for refractory GVHD (Bloor et al., 2020). The iPSC-MSC approach serves as an excellent solution for scaling MSC manufacturing without sacrificing therapeutic potency through the passage and expansion of cells. One major concern regarding iPSC-MSCs is that the viral vector-based strategy for reprogramming might present a potential for tumorigenic transformation. However, recent developments in non-viral-based technologies, such as non-integrating episomal plasmid-based reprogramming (Slamecka et al., 2016), might present safer strategies for the generation of iPSC-MSCs suitable for use in a clinical setting (Sabapathy and Kumar, 2016). Human embryonic stem cells and iPSCs have already been extensively investigated for use as potential retinal stem cell treatments (Takagi et al., 2019); GMP-grade iPSC-MSCs may improve the efficacy of cell therapy for retinal degeneration.

Another strategy to overcome MSC product heterogeneity is using primed MSCs or boosting the innate therapeutic efficacy by other bioengineering methods (Levy et al., 2020). For instance, using a medium-based approach (similar to pre-licensing), MSCs can be induced to secrete elevated levels of neurotropic factors, including GDNF, BDNF, VEGF, and HGF, which have been shown to have protective effects in animal models of neurodegenerative diseases (Gothelf et al., 2014). When administered to patients with neurodegenerative diseases, these primed MSCs have been demonstrated to simultaneously deliver multiple neurotrophins and immunomodulatory components (Gothelf et al., 2017). While some pre-clinical studies have shown promising results of primed MSCs for RDDs, such as erythropoietin (EPO)-expressed rat MSCs (Guan et al., 2013), BDNF-expressed MSCs (Levkovitch-Verbin et al., 2010; Harper et al., 2011), it remains to be seen whether or not these engineered MSCs improve therapeutic outcomes in a clinical setting.

### Optimization of Delivery Methods

A pre-clinical study indicated that systemically administered MSCs did not migrate to the injured eyes and had no effects on RDDs, as they are trapped in lung capillary beds (Johnson et al., 2010; Ge et al., 2014). Thus, local administration is the major route of delivery of MSCs for RDD therapy, as this route can deliver paracrine factors directly to the retina. Most clinical trials use intravitreal or subretinal transplantation, but subtenon's or retrobulbar injections are also used in the non-FDA registered, patient-funded clinical trial “Stem cell ophthalmology treatment study (SCOTS)” (Singh et al., 2020). It is imperative that the transplantation techniques have a reasonably low risk, so that the safety and efficacy of the MSCs can be evaluated properly.

Subretinal injection allows direct contact of the host and MSCs, but the injection procedure may disturb the retina. There are two main surgical approaches, internal and external. The internal approach requires an initial vitrectomy, after which a small-gauge needle is introduced into the eye via the pars plana and is passed through a retinotomy at the injection site and the cell suspension is deposited in the subretinal space near the fovea. The external approach involves subretinal delivery of cells via a cannula delivered outside the eye through the suprachoroidal space. This technique avoids exposure of the cells to the vitreous cavity but can induce retinal perforation and detachment (Ho et al., 2017). These severe adverse effects (retinal detachment or perforation) can be avoided by optimizing the procedures, as shown in a phase 2b study (Heier et al., 2020).

The intravitreal delivery approach is straightforward and widely used in anti-VEGF therapy in the ophthalmic clinics. The MSCs are injected as a suspension into the vitreous cavity via a needle through the pars plana. The cells do not gain access to the subretinal space and remain in the vitreous. Cellular cluster formation in the vitreous occurred in some cases of MSC intravitreal injection (Tzameret et al., 2014). As MSCs generally do not integrate into the retina, intravitreal injection is safer than subretinal injection. However, MSCs injected into vitreous cavity can still cause secondary glaucoma, epiretinal membrane and PVR (Kuriyan et al., 2017; Park et al., 2017; Khine et al., 2020). MSCs may locate on the retinal surface and differentiate improperly into myofibroblast-like cells which may induce fibrosis, PVR, and tractional retinal detachment; they can also increase oxygen demand, inducing ischemic microvascular changes and subsequent ocular neovascularization (Kuriyan et al., 2017; Khine et al., 2020). There are no definitive answers about which delivery approach leads to the best therapeutic effect. Thus, these surgery-related severe adverse effects will need to be carefully monitored in clinical trials.

Both intravitreal and subretinal injection can disturb the host BRB and expose the grafted cells to the host retinal immune system and thus raise concerns about long-term donor cell survival. Indeed, both the retention and survival of MSCs following local administration are important factors affecting the therapeutic outcome (Levy et al., 2020), despite pre-clinical study suggesting that the therapeutic potency to protect the retinal cells is not dependent upon the long-term survival of the donor cells (Lu et al., 2010). Nevertheless, multiple strategies have been investigated to improve the local administration of MSCs, including priming MSCs *in vitro* (Levy et al., 2020). For example, hypoxic priming upregulated the expression of prosurvival factors such as hypoxia-inducible factor 1, which can help MSCs adapt to the typically hypoxic disease site (Hu et al., 2008). Similarly, human MSCs from Wharton's jelly (hWJMSCs) expressing erythropoietin enhance the survival of retinal neurons against oxidative stress (Shirley Ding et al., 2018). Using biomaterials to encapsulate MSCs is another promising strategy to overcome challenges associated with local administration (Führmann et al., 2016). For example, HGF-overexpressing MSCs in a synthetic peptide-based hydrogel survived much longer and showed superior reduction in scar formation compared with native MSCs (Wu et al., 2017). It will be interesting to test these strategies in the MSC clinical trials for RDDs treatment in the future.

## Conclusions

Retinal degeneration diseases are a leading cause of blindness worldwide and there is no effective treatment. MSCs can be easily isolated from multiple tissues and have shown promise in treating many diseases by restoring organ homeostasis in inflamed, injured, or diseased tissues. Pre-clinical animal studies suggested that their trophic and immunomodulatory properties can protect retinal neurons and enhance the function of retinal pigment epithelium cells from an array of retinal degeneration diseases. Recent clinical trials have demonstrated promising safety profiles for the application of MSCs in retinal diseases and have provided valuable data for future exploration. However, significant complications that arise from poorly designed clinical trials, questionable practices or regulatory shortcuts could substantially hinder research in MSC-based retinal therapies. Nevertheless, MSCs have great therapeutic potential for the treatment of retinal degeneration. A better understanding of the interaction between MSCs and host retinal degenerative environments is the key to yield an optimal benefit. Overcoming the heterogeneity in the MSC product and optimizing ocular surgical delivery to avoid adverse sequela are major challenges to translational use of MSCs to treat retinal degeneration.

## Author Contributions

YL and DC were involved in the concept and design. YL and XR wrote the first version of the manuscript. YL, YC, and DC reviewed and revised the manuscript. All authors contributed to the article and approved the submitted version.

## Conflict of Interest

The authors declare that the research was conducted in the absence of any commercial or financial relationships that could be construed as a potential conflict of interest.

## References

[B1] Aharony I. Michowiz S. Goldenberg-Cohen N. (2017). The promise of stem cell-based therapeutics in ophthalmology. Neural Regen. Res. 12, 173–180. 10.4103/1673-5374.200793 28400789 PMC5361491

[B2] Akhtar-Schafer I. Wang L. Krohne T. U. Xu H. Langmann T. (2018). Modulation of three key innate immune pathways for the most common retinal degenerative diseases. EMBO Mol. Med. 10:e8259 10.15252/emmm.201708259 30224384 PMC6180304

[B3] Anderson J. D. Johansson H. J. Graham C. S. Vesterlund M. Pham M. T. Bramlett C. S.. (2016). Comprehensive proteomic analysis of mesenchymal stem cell exosomes reveals modulation of angiogenesis via nuclear factor-KappaB signaling. Stem Cells 34, 601–613. 10.1002/stem.2298 26782178 PMC5785927

[B4] Ankrum J. A. Dastidar R. G. Ong J. F. Levy O. Karp J. M. (2014a). Performance-enhanced mesenchymal stem cells via intracellular delivery of steroids. Sci. Rep. 4:4645. 10.1038/srep04645 24717973 PMC3982175

[B5] Ankrum J. A. Ong J. F. Karp J. M. (2014b). Mesenchymal stem cells: immune evasive, not immune privileged. Nat. Biotechnol. 32, 252–260. 10.1038/nbt.2816 24561556 PMC4320647

[B6] Apatoff M. B. L. Sengillo J. D. White E. C. Bakhoum M. F. Bassuk A. G. Mahajan V. B.. (2018). Autologous stem cell therapy for inherited and acquired retinal disease. Regen. Med. 13, 89–96. 10.2217/rme-2017-0089 29360008 PMC6123878

[B7] Arnhold S. Absenger Y. Klein H. Addicks K. Schraermeyer U. (2007). Transplantation of bone marrow-derived mesenchymal stem cells rescue photoreceptor cells in the dystrophic retina of the rhodopsin knockout mouse. Graefes Arch. Clin. Exp. Ophthalmol. 245, 414–422. 10.1007/s00417-006-0382-7 16896916

[B8] Arroba A. I. Campos-Caro A. Aguilar-Diosdado M. Valverde A. M. (2018). IGF-1, inflammation and retinal degeneration: a close network. Front. Aging Neurosci 10:203. 10.3389/fnagi.2018.00203 30026694 PMC6041402

[B9] Bloom D. D. Centanni J. M. Bhatia N. Emler C. A. Drier D. Leverson G. E.. (2015). A reproducible immunopotency assay to measure mesenchymal stromal cell-mediated T-cell suppression. Cytotherapy 17, 140–151. 10.1016/j.jcyt.2014.10.002 25455739 PMC4297551

[B10] Bloor A. J. C. Patel A. Griffin J. E. Gilleece M. H. Radia R. Yeung D. T.. (2020). Production, safety and efficacy of iPSC-derived mesenchymal stromal cells in acute steroid-resistant graft versus host disease: a phase I, multicenter, open-label, dose-escalation study. Nat. Med. 26, 1720–1725. 10.1038/s41591-020-1050-x 32929265

[B11] Boland L. Burand A. J. Brown A. J. Boyt D. Lira V. A. Ankrum J. A. (2018). IFN-γ and TNF-α Pre-licensing protects mesenchymal stromal cells from the pro-inflammatory effects of palmitate. Mol. Ther. 26, 860–873. 10.1016/j.ymthe.2017.12.013 29352647 PMC5910660

[B12] Boudreault K. Justus S. Lee W. Mahajan V. B. Tsang S. H. (2016). Complication of autologous stem cell transplantation in retinitis pigmentosa. JAMA Ophthalmol. 134, 711–712. 10.1001/jamaophthalmol.2016.0803 27149677

[B13] Boyt D. T. Boland L. K. Burand A. J. Jr. Brown A. J. Ankrum J. A. (2020). Dose and duration of interferon γ pre-licensing interact with donor characteristics to influence the expression and function of indoleamine-2,3-dioxygenase in mesenchymal stromal cells. J. R. Soc. Interface 17:20190815. 10.1098/rsif.2019.0815 32546114 PMC7328385

[B14] Burand A. J. Gramlich O. W. Brown A. J. Ankrum J. A. (2017). Function of cryopreserved mesenchymal stromal cells with and without interferon-γ prelicensing is context dependent. Stem Cells 35, 1437–1439. 10.1002/stem.2528 27758056 PMC5397371

[B15] Cao J. Murat C. An W. Yao X. Lee J. Santulli-Marotto S.. (2016). Human umbilical tissue-derived cells rescue retinal pigment epithelium dysfunction in retinal degeneration. Stem Cells 34, 367–379. 10.1002/stem.2239 26523756

[B16] Çerman E. Akkoç T. Eraslan M. Sahin zkara S. Vardar Aker F. Subaşi C. (2016). Retinal electrophysiological effects of intravitreal bone marrow derived mesenchymal stem cells in streptozotocin induced diabetic rats. PLoS ONE 11:e0156495 10.1371/journal.pone.0156495 27300133 PMC4907488

[B17] Cotrim C. C. Toscano L. Messias A. Jorge R. Siqueira R. C. (2017). Intravitreal use of bone marrow mononuclear fraction containing CD34(+) stem cells in patients with atrophic age-related macular degeneration. Clin. Ophthalmol. 11, 931–938. 10.2147/OPTH.S133502 28579742 PMC5449098

[B18] Court A. C. Le-Gatt A. Luz-Crawford P. Parra E. Aliaga-Tobar V. Batiz L. F.. (2020). Mitochondrial transfer from MSCs to T cells induces Treg differentiation and restricts inflammatory response. EMBO Rep. 21:e48052. 10.15252/embr.201948052 31984629 PMC7001501

[B19] Dalkara D. Goureau O. Marazova K. Sahel J. A. (2016). Let there be light: gene and cell therapy for blindness. Hum. Gene Ther. 27, 134–147. 10.1089/hum.2015.147 26751519 PMC4779297

[B20] De Witte S. F. H. Luk F. Sierra Parraga J. M. Gargesha M. Merino A. Korevaar S. S.. (2018). Immunomodulation by therapeutic mesenchymal stromal cells (MSC) is triggered through phagocytosis of MSC by monocytic cells. Stem Cells 36, 602–615. 10.1002/stem.2779 29341339

[B21] Ding D. C. Shyu W. C. Lin S. Z. (2011). Mesenchymal stem cells. Cell Transplant 20, 5–14. 10.3727/096368910X 21396235

[B22] Ding S. L. S. Kumar S. Mok P. L. (2017). Cellular reparative mechanisms of mesenchymal stem cells for retinal diseases. Int. J. Mol. Sci. 18:1406. 10.3390/ijms18081406 28788088 PMC5577990

[B23] Dominici M. Le Blanc K. Mueller I. Slaper-Cortenbach I. Marini F. Krause D.. (2006). Minimal criteria for defining multipotent mesenchymal stromal cells. The International Society for Cellular Therapy position statement. Cytotherapy 8, 315–317. 10.1080/14653240600855905 16923606

[B24] Elshaer S. L. Evans W. Pentecost M. Lenin R. Periasamy R. Jha K. A.. (2018). Adipose stem cells and their paracrine factors are therapeutic for early retinal complications of diabetes in the Ins2(Akita) mouse. Stem Cell Res. Ther. 9:322. 10.1186/s13287-018-1059-y 30463601 PMC6249931

[B25] Enzmann V. Lecaude S. Kruschinski A. Vater A. (2017). CXCL12/SDF-1-dependent retinal migration of endogenous bone marrow-derived stem cells improves visual function after pharmacologically induced retinal degeneration. Stem Cell Rev. Rep. 13, 278–286. 10.1007/s12015-016-9706-0 27924617

[B26] Ezquer M. Urzua C. A. Montecino S. Leal K. Conget P. Ezquer F. (2016). Intravitreal administration of multipotent mesenchymal stromal cells triggers a cytoprotective microenvironment in the retina of diabetic mice. Stem Cell Res. Ther. 7:42. 10.1186/s13287-016-0299-y 26983784 PMC4793534

[B27] Ferrara N. Adamis A. P. (2016). Ten years of anti-vascular endothelial growth factor therapy. Nat. Rev. Drug Discov 15, 385–403. 10.1038/nrd.2015.17 26775688

[B28] Francis P. J. Wang S. Zhang Y. Brown A. Hwang T. Mcfarland T. J.. (2009). Subretinal transplantation of forebrain progenitor cells in nonhuman primates: survival and intact retinal function. Invest. Ophthalmol. Vis. Sci. 50, 3425–3431. 10.1167/iovs.08-2908 19234356 PMC2826708

[B29] Fričová D. Korchak J. A. Zubair A. C. (2020). Challenges and translational considerations of mesenchymal stem/stromal cell therapy for Parkinson's disease. NPJ Regen. Med. 5, 1–10. 10.1038/s41536-020-00106-y 33298940 PMC7641157

[B30] Führmann T. Tam R. Y. Ballarin B. Coles B. Elliott Donaghue I. Van Der Kooy D.. (2016). Injectable hydrogel promotes early survival of induced pluripotent stem cell-derived oligodendrocytes and attenuates longterm teratoma formation in a spinal cord injury model. Biomaterials 83, 23–36. 10.1016/j.biomaterials.2015.12.032 26773663

[B31] Fukuda S. Nagano M. Yamashita T. Kimura K. Tsuboi I. Salazar G.. (2013). Functional endothelial progenitor cells selectively recruit neurovascular protective monocyte-derived F4/80(+) /Ly6c(+) macrophages in a mouse model of retinal degeneration. Stem Cells 31, 2149–2161. 10.1002/stem.1469 23843337

[B32] Galipeau J. Sensébé L. (2018). mesenchymal stromal cells: clinical challenges and therapeutic opportunities. Cell Stem Cell 22, 824–833. 10.1016/j.stem.2018.05.004 29859173 PMC6434696

[B33] Galleu A. Riffo-Vasquez Y. Trento C. Lomas C. Dolcetti L. Cheung T. S.. (2017). Apoptosis in mesenchymal stromal cells induces *in vivo* recipient-mediated immunomodulation. Sci. Transl. Med. 9:eaam7828. 10.1126/scitranslmed.aam7828 29141887

[B34] Ge J. Guo L. Wang S. Zhang Y. Cai T. Zhao R. C.. (2014). The size of mesenchymal stem cells is a significant cause of vascular obstructions and stroke. Stem Cell Rev. Rep. 10, 295–303. 10.1007/s12015-013-9492-x 24390934

[B35] Ghosh F. Abdshill H. Arner K. Voss U. Taylor L. (2018). Retinal neuroinflammatory induced neuronal degeneration - role of toll-like receptor-4 and relationship with gliosis. Exp. Eye Res. 169, 99–110. 10.1016/j.exer.2018.02.002 29425879

[B36] Gorbatyuk M. Gorbatyuk O. (2013). Review: retinal degeneration: focus on the unfolded protein response. Mol. Vis. 19, 1985–1998. Available online at: http://www.molvis.org/molvis/v19/1985/24068865 PMC3782367

[B37] Gothelf Y. Abramov N. Harel A. Offen D. (2014). Safety of repeated transplantations of neurotrophic factors-secreting human mesenchymal stromal stem cells. Clin. Transl. Med. 3:21. 10.1186/2001-1326-3-21 25097724 PMC4108239

[B38] Gothelf Y. Kaspi H. Abramov N. Aricha R. (2017). miRNA profiling of NurOwn®: mesenchymal stem cells secreting neurotrophic factors. Stem Cell Res. Ther. 8:249. 10.1186/s13287-017-0692-1 29116031 PMC5678806

[B39] Gu X. Yu X. Zhao C. Duan P. Zhao T. Liu Y.. (2018). Efficacy and safety of autologous bone marrow mesenchymal stem cell transplantation in patients with diabetic retinopathy. Cell. Physiol. Biochem. 49, 40–52. 10.1159/000492838 30134223

[B40] Guan Y. Cui L. Qu Z. Lu L. Wang F. Wu Y.. (2013). Subretinal transplantation of rat MSCs and erythropoietin gene modified rat MSCs for protecting and rescuing degenerative retina in rats. Curr. Mol. Med. 13, 1419–1431. 10.2174/15665240113139990071 23971737

[B41] Han X. Yang Q. Lin L. Xu C. Zheng C. Chen X.. (2014). Interleukin-17 enhances immunosuppression by mesenchymal stem cells. Cell Death Differ. 21, 1758–1768. 10.1038/cdd.2014.85 25034782 PMC4211372

[B42] Harper M. M. Grozdanic S. D. Blits B. Kuehn M. H. Zamzow D. Buss J. E.. (2011). Transplantation of BDNF-secreting mesenchymal stem cells provides neuroprotection in chronically hypertensive rat eyes. Invest. Ophthalmol. Vis. Sci. 52, 4506–4515. 10.1167/iovs.11-7346 21498611 PMC3175938

[B43] Harrell C. R. Fellabaum C. Arsenijevic A. Markovic B. S. Djonov V. Volarevic V. (2019). Therapeutic potential of mesenchymal stem cells and their secretome in the treatment of glaucoma. Stem Cells Int. 2019:7869130. 10.1155/2019/7869130 31949441 PMC6948292

[B44] Heier J. S. Ho A. C. Samuel M. A. Chang T. Riemann C. D. Kitchens J. W.. (2020). Safety and efficacy of subretinally administered palucorcel for geographic atrophy of age-related macular degeneration: phase 2b study. Ophthalmol. Retina 4, 384–393. 10.1016/j.oret.2019.11.011 32033908

[B45] Hemeda H. Jakob M. Ludwig A. K. Giebel B. Lang S. Brandau S. (2010). Interferon-gamma and tumor necrosis factor-alpha differentially affect cytokine expression and migration properties of mesenchymal stem cells. Stem Cells Dev. 19, 693–706. 10.1089/scd.2009.0365 20067407

[B46] Hermankova B. Kossl J. Bohacova P. Javorkova E. Hajkova M. Krulova M.. (2019). The immunomodulatory potential of mesenchymal stem cells in a retinal inflammatory environment. Stem Cell Rev. Rep. 15, 880–891. 10.1007/s12015-019-09908-0 31863334

[B47] Ho A. C. Chang T. S. Samuel M. Williamson P. Willenbucher R. F. Malone T. (2017). Experience with a subretinal cell-based therapy in patients with geographic atrophy secondary to age-related macular degeneration. Am. J. Ophthalmol. 179, 67–80. 10.1016/j.ajo.2017.04.006 28435054

[B48] Holan V. Hermankova B. Krulova M. Zajicova A. (2019). Cytokine interplay among the diseased retina, inflammatory cells and mesenchymal stem cells - a clue to stem cell-based therapy. World J. Stem Cells 11, 957–967. 10.4252/wjsc.v11.i11.957 31768222 PMC6851013

[B49] Hu X. Yu S. P. Fraser J. L. Lu Z. Ogle M. E. Wang J. A.. (2008). Transplantation of hypoxia-preconditioned mesenchymal stem cells improves infarcted heart function via enhanced survival of implanted cells and angiogenesis. J. Thorac. Cardiovasc. Surg. 135, 799–808. 10.1016/j.jtcvs.2007.07.071 18374759

[B50] Huang H. Kolibabka M. Eshwaran R. Chatterjee A. Schlotterer A. Willer H.. (2019). Intravitreal injection of mesenchymal stem cells evokes retinal vascular damage in rats. FASEB J. 33, 14668–14679. 10.1096/fj.201901500R 31690119

[B51] Huang L. Li Z. Tian H. Wang W. Cui D. Zhou Z.. (2017). Adult human periodontal ligament-derived stem cells delay retinal degeneration and maintain retinal function in RCS rats. Stem Cell Res. Ther. 8, 290. 10.1186/s13287-017-0731-y 29273085 PMC5741902

[B52] Hwang J. C. Staropoli P. C. Kuriyan A. E. Yannuzzi N. A. Sridhar J. (2020). Stem cell therapy, ophthalmic applications, and the current controversies with direct-to-consumer marketing. Int. Ophthalmol. Clin. 60, 179–192. 10.1097/IIO.0000000000000329 33093325

[B53] Inana G. Murat C. An W. Yao X. Harris I. R. Cao J. (2018). RPE phagocytic function declines in age-related macular degeneration and is rescued by human umbilical tissue derived cells. J. Transl. Med. 16:63. 10.1186/s12967-018-1434-6 29534722 PMC5851074

[B54] Inoue Y. Iriyama A. Ueno S. Takahashi H. Kondo M. Tamaki Y.. (2007). Subretinal transplantation of bone marrow mesenchymal stem cells delays retinal degeneration in the RCS rat model of retinal degeneration. Exp. Eye Res. 85, 234–241. 10.1016/j.exer.2007.04.007 17570362

[B55] Jiang D. Gao F. Zhang Y. Wong D. S. Li Q. Tse H. F.. (2016). Mitochondrial transfer of mesenchymal stem cells effectively protects corneal epithelial cells from mitochondrial damage. Cell Death Dis. 7:e2467. 10.1038/cddis.2016.358 27831562 PMC5260876

[B56] Jiang D. Xiong G. Feng H. Zhang Z. Chen P. Yan B.. (2019). Donation of mitochondria by iPSC-derived mesenchymal stem cells protects retinal ganglion cells against mitochondrial complex I defect-induced degeneration. Theranostics 9, 2395–2410. 10.7150/thno.29422 31149051 PMC6531297

[B57] Jiang L. Q. Jorquera M. Streilein J. W. (1993). Subretinal space and vitreous cavity as immunologically privileged sites for retinal allografts. Invest. Ophthalmol. Vis. Sci. 34, 3347–3354. 8225870

[B58] Jin Z. B. Gao M. L. Deng W. L. Wu K. C. Sugita S. Mandai M.. (2019). Stemming retinal regeneration with pluripotent stem cells. Prog. Retin. Eye Res. 69, 38–56. 10.1016/j.preteyeres.2018.11.003 30419340

[B59] Johnson T. V. Bull N. D. Hunt D. P. Marina N. Tomarev S. I. Martin K. R. (2010). Neuroprotective effects of intravitreal mesenchymal stem cell transplantation in experimental glaucoma. Invest. Ophthalmol. Vis. Sci. 51, 2051–2059. 10.1167/iovs.09-4509 19933193 PMC2868400

[B60] Kauppinen A. Paterno J. J. Blasiak J. Salminen A. Kaarniranta K. (2016). Inflammation and its role in age-related macular degeneration. Cell. Mol. Life Sci. 73, 1765–1786. 10.1007/s00018-016-2147-8 26852158 PMC4819943

[B61] Khine K. T. Albini T. A. Lee R. K. (2020). Chronic retinal detachment and neovascular glaucoma after intravitreal stem cell injection for Usher Syndrome. Am. J. Ophthalmol. Case Rep. 18:100647. 10.1016/j.ajoc.2020.100647 32211560 PMC7082495

[B62] Kocan B. Maziarz A. Tabarkiewicz J. Ochiya T. Banas-Zabczyk A. (2017). Trophic activity and phenotype of adipose tissue-derived mesenchymal stem cells as a background of their regenerative potential. Stem Cells Int. 2017:1653254. 10.1155/2017/1653254 28757877 PMC5516761

[B63] Kuriyan A. E. Albini T. A. Townsend J. H. Rodriguez M. Pandya H. K. Leonard R. E. 2nd Parrott M. B.. (2017). Vision loss after intravitreal injection of autologous “stem cells” for AMD. N. Engl. J. Med. 376, 1047–1053. 10.1056/NEJMoa1609583 28296617 PMC5551890

[B64] Labrador-Velandia S. Alonso-Alonso M. L. Di Lauro S. Garcia-Gutierrez M. T. Srivastava G. K. Pastor J. C.. (2019). Mesenchymal stem cells provide paracrine neuroprotective resources that delay degeneration of co-cultured organotypic neuroretinal cultures. Exp. Eye Res. 185:107671. 10.1016/j.exer.2019.05.011 31108056

[B65] Leung E. H. Flynn H. W. Jr. Albini T. A. Medina C. A. (2016). Retinal Detachment After Subretinal Stem Cell Transplantation. Ophthalmic Surg. Lasers Imaging Retina 47, 600–601. 10.3928/23258160-20160601-16 27327294

[B66] Levkovitch-Verbin H. Sadan O. Vander S. Rosner M. Barhum Y. Melamed E.. (2010). Intravitreal injections of neurotrophic factors secreting mesenchymal stem cells are neuroprotective in rat eyes following optic nerve transection. Invest. Ophthalmol. Vis. Sci. 51, 6394–6400. 10.1167/iovs.09-4310 20926814

[B67] Levy O. Kuai R. Siren E. M. J. Bhere D. Milton Y. Nissar N.. (2020). Shattering barriers toward clinically meaningful MSC therapies. Sci. Adv. 6:eaba6884. 10.1126/sciadv.aba6884 32832666 PMC7439491

[B68] Li N. Li X. R. Yuan J. Q. (2009). Effects of bone-marrow mesenchymal stem cells transplanted into vitreous cavity of rat injured by ischemia/reperfusion. Graefes Arch. Clin. Exp. Ophthalmol. 247, 503–514. 10.1007/s00417-008-1009-y 19084985

[B69] Li T. Xia M. Gao Y. Chen Y. Xu Y. (2015). Human umbilical cord mesenchymal stem cells: an overview of their potential in cell-based therapy. Expert Opin. Biol. Ther. 15, 1293–1306. 10.1517/14712598.2015.1051528 26067213

[B70] Li Z. Wang J. Gao F. Zhang J. Tian H. Shi X.. (2016a). Human adipose-derived stem cells delay retinal degeneration in royal college of surgeons rats through anti-apoptotic and VEGF-mediated neuroprotective effects. Curr. Mol. Med. 16, 553–566. 10.2174/1566524016666160607090538 27280496

[B71] Li Z. Zeng Y. Chen X. Li Q. Wu W. Xue L.. (2016b). Neural stem cells transplanted to the subretinal space of rd1 mice delay retinal degeneration by suppressing microglia activation. Cytotherapy 18, 771–784. 10.1016/j.jcyt.2016.03.001 27067610

[B72] Liu H. Li D. Zhang Y. Li M. (2018). Inflammation, mesenchymal stem cells and bone regeneration. Histochem. Cell Biol. 149, 393–404. 10.1007/s00418-018-1643-3 29435765

[B73] Liu T. Zhang L. Joo D. Sun S. C. (2017). NF-κB signaling in inflammation. Signal Transduct Target Ther. 2, 17023-. 10.1038/sigtrans.2017.23 29158945 PMC5661633

[B74] Lu B. Wang S. Girman S. Mcgill T. Ragaglia V. Lund R. (2010). Human adult bone marrow-derived somatic cells rescue vision in a rodent model of retinal degeneration. Exp. Eye Res. 91, 449–455. 10.1016/j.exer.2010.06.024 20603115

[B75] Ludwig P. E. Freeman S. C. Janot A. C. (2019). Novel stem cell and gene therapy in diabetic retinopathy, age related macular degeneration, and retinitis pigmentosa. Int J Retina Vitreous 5:7. 10.1186/s40942-019-0158-y 30805203 PMC6373096

[B76] Lund R. D. Wang S. Lu B. Girman S. Holmes T. Sauvé Y.. (2007). Cells isolated from umbilical cord tissue rescue photoreceptors and visual functions in a rodent model of retinal disease. Stem Cells. 25, 602–611. 10.1634/stemcells.2006-0308erratum 17053209

[B77] Marino L. Castaldi M. A. Rosamilio R. Ragni E. Vitolo R. Fulgione C.. (2019). Mesenchymal stem cells from the wharton's jelly of the human umbilical cord: biological properties and therapeutic potential. Int. J. Stem Cells 12, 218–226. 10.15283/ijsc18034 31022994 PMC6657936

[B78] Mastri M. Shah Z. Mclaughlin T. Greene C. J. Baum L. Suzuki G.. (2012). Activation of Toll-like receptor 3 amplifies mesenchymal stem cell trophic factors and enhances therapeutic potency. Am. J. Physiol. Cell Physiol. 303, C1021–1033. 10.1152/ajpcell.00191.2012 22843797 PMC3492833

[B79] Mathew B. Ravindran S. Liu X. Torres L. Chennakesavalu M. Huang C. C.. (2019). Mesenchymal stem cell-derived extracellular vesicles and retinal ischemia-reperfusion. Biomaterials 197, 146–160. 10.1016/j.biomaterials.2019.01.016 30654160 PMC6425741

[B80] Mclelland B. T. Lin B. Mathur A. Aramant R. B. Thomas B. B. Nistor G.. (2018). Transplanted hESC-derived retina organoid sheets differentiate, integrate, and improve visual function in retinal degenerate rats. Invest. Ophthalmol. Vis. Sci. 59, 2586–2603. 10.1167/iovs.17-23646 29847666 PMC5968836

[B81] Mead B. Berry M. Logan A. Scott R. A. Leadbeater W. Scheven B. A. (2015). Stem cell treatment of degenerative eye disease. Stem Cell Res. 14, 243–257. 10.1016/j.scr.2015.02.003 25752437 PMC4434205

[B82] Mead B. Hill L. J. Blanch R. J. Ward K. Logan A. Berry M.. (2016). Mesenchymal stromal cell-mediated neuroprotection and functional preservation of retinal ganglion cells in a rodent model of glaucoma. Cytotherapy 18, 487–496. 10.1016/j.jcyt.2015.12.002 26897559

[B83] Mead B. Logan A. Berry M. Leadbeater W. Scheven B. A. (2013). Intravitreally transplanted dental pulp stem cells promote neuroprotection and axon regeneration of retinal ganglion cells after optic nerve injury. Invest. Ophthalmol. Vis. Sci. 54, 7544–7556. 10.1167/iovs.13-13045 24150755

[B84] Mead B. Logan A. Berry M. Leadbeater W. Scheven B. A. (2017). Concise review: dental pulp stem cells: a novel cell therapy for retinal and central nervous system repair. Stem Cells 35, 61–67. 10.1002/stem.2398 27273755

[B85] Mead B. Tomarev S. (2017). Bone marrow-derived mesenchymal stem cells-derived exosomes promote survival of retinal ganglion cells through miRNA-dependent mechanisms. Stem Cells Transl. Med. 6, 1273–1285. 10.1002/sctm.16-0428 28198592 PMC5442835

[B86] Melief S. M. Zwaginga J. J. Fibbe W. E. Roelofs H. (2013). Adipose tissue-derived multipotent stromal cells have a higher immunomodulatory capacity than their bone marrow-derived counterparts. Stem Cells Transl. Med. 2, 455–463. 10.5966/sctm.2012-0184 23694810 PMC3673757

[B87] Mesquida M. Drawnel F. Fauser S. (2019). The role of inflammation in diabetic eye disease. Semin. Immunopathol. 41, 427–445. 10.1007/s00281-019-00750-7 31175392

[B88] Millán-Rivero J. E. Nadal-Nicolás F. M. García-Bernal D. Sobrado-Calvo P. Blanquer M. Moraleda J. M.. (2018). Human Wharton's jelly mesenchymal stem cells protect axotomized rat retinal ganglion cells via secretion of anti-inflammatory and neurotrophic factors. Sci. Rep. 8:16299. 10.1038/s41598-018-34527-z 30389962 PMC6214908

[B89] Mugisho O. O. Rupenthal I. D. Squirrell D. M. Bould S. J. Danesh-Meyer H. V. Zhang J.. (2018). Intravitreal pro-inflammatory cytokines in non-obese diabetic mice: Modelling signs of diabetic retinopathy. PLoS ONE 13:e0202156. 10.1371/journal.pone.0202156 30133488 PMC6105000

[B90] Murphy M. B. Moncivais K. Caplan A. I. (2013). Mesenchymal stem cells: environmentally responsive therapeutics for regenerative medicine. Exp. Mol. Med. 45, e54–e54. 10.1038/emm.2013.94 24232253 PMC3849579

[B91] Murray I. R. West C. C. Hardy W. R. James A. W. Park T. S. Nguyen A.. (2014). Natural history of mesenchymal stem cells, from vessel walls to culture vessels. Cell. Mol. Life Sci. 71, 1353–1374. 10.1007/s00018-013-1462-6 24158496 PMC11113613

[B92] Mutt S. J. Karhu T. Lehtonen S. Lehenkari P. Carlberg C. Saarnio J. (2012). Inhibition of cytokine secretion from adipocytes by 1,25-dihydroxyvitamin D3 via the NF-κB pathway. FASEB J. 26, 4400–4407. 10.1096/fj.12-210880 22798425

[B93] Naji A. Eitoku M. Favier B. Deschaseaux F. Rouas-Freiss N. Suganuma N. (2019). Biological functions of mesenchymal stem cells and clinical implications. Cell. Mol. Life Sci. 76, 3323–3348. 10.1007/s00018-019-03125-1 31055643 PMC11105258

[B94] Nakano M. Nagaishi K. Konari N. Saito Y. Chikenji T. Mizue Y.. (2016). Bone marrow-derived mesenchymal stem cells improve diabetes-induced cognitive impairment by exosome transfer into damaged neurons and astrocytes. Sci. Rep. 6:24805. 10.1038/srep24805 27102354 PMC4840335

[B95] Nirwan R. S. Albini T. A. Sridhar J. Flynn H. W. Jr. Kuriyan A. E. (2019). Assessing “cell therapy” clinics offering treatments of ocular conditions using direct-to-consumer marketing websites in the United States. Ophthalmology 126, 1350–1355. 10.1016/j.ophtha.2019.03.019 30904542 PMC6754792

[B96] Oner A. Gonen Z. B. Sevim D. G. Smim Kahraman N. Unlu M. (2018). Suprachoroidal adipose tissue-derived mesenchymal stem cell implantation in patients with dry-type age-related macular degeneration and stargardt's macular dystrophy: 6-month follow-up results of a phase 2 study. Cell. Reprogram 20, 329–336. 10.1089/cell.2018.0045 31251672

[B97] Oner A. Gonen Z. B. Sinim N. Cetin M. Ozkul Y. (2016). Subretinal adipose tissue-derived mesenchymal stem cell implantation in advanced stage retinitis pigmentosa: a phase I clinical safety study. Stem Cell Res. Ther. 7:178. 10.1186/s13287-016-0432-y 27906070 PMC5134260

[B98] Oswald J. Baranov P. (2018). Regenerative medicine in the retina: from stem cells to cell replacement therapy. Ther. Adv. Ophthalmol. 10:2515841418774433. 10.1177/2515841418774433 29998222 PMC6016968

[B99] Otani A. Dorrell M. I. Kinder K. Moreno S. K. Nusinowitz S. Banin E.. (2004). Rescue of retinal degeneration by intravitreally injected adult bone marrow-derived lineage-negative hematopoietic stem cells. J. Clin. Invest. 114, 765–774. 10.1172/JCI200421686 15372100 PMC516263

[B100] Ozay E. I. Vijayaraghavan J. Gonzalez-Perez G. Shanthalingam S. Sherman H. L. Garrigan D. T. Jr.. (2019). Cymerus™ iPSC-MSCs significantly prolong survival in a pre-clinical, humanized mouse model of Graft-vs-host disease. Stem Cell Res. 35:101401. 10.1016/j.scr.2019.101401 30738321 PMC6544140

[B101] Park S. S. Moisseiev E. Bauer G. Anderson J. D. Grant M. B. Zam A.. (2017). Advances in bone marrow stem cell therapy for retinal dysfunction. Prog. Retin. Eye Res. 56, 148–165. 10.1016/j.preteyeres.2016.10.002 27784628 PMC5237620

[B102] Poon M. W. Yan L. Jiang D. Qin P. Tse H. F. Wong I. Y.. (2015). Inhibition of RAP1 enhances corneal recovery following alkali injury. Invest. Ophthalmol. Vis. Sci. 56, 711–721. 10.1167/iovs.14-15268 25574050

[B103] Rajashekhar G. Ramadan A. Abburi C. Callaghan B. Traktuev D. O. Evans-Molina C.. (2014). Regenerative therapeutic potential of adipose stromal cells in early stage diabetic retinopathy. PLoS ONE 9:e84671. 10.1371/journal.pone.0084671 24416262 PMC3886987

[B104] Rashid K. Wolf A. Langmann T. (2018). Microglia activation and immunomodulatory therapies for retinal degenerations. Front. Cell. Neurosci. 12:176. 10.3389/fncel.2018.00176 29977192 PMC6021747

[B105] Ren G. Su J. Zhang L. Zhao X. Ling W. L'huillie A.. (2009). Species variation in the mechanisms of mesenchymal stem cell-mediated immunosuppression. Stem Cells 27, 1954–1962. 10.1002/stem.118 19544427

[B106] Rong A. J. Lam B. L. Ansari Z. A. Albini T. A. (2018). Vision loss secondary to autologous adipose stem cell injections: a rising problem. JAMA Ophthalmol. 136, 97–99. 10.1001/jamaophthalmol.2017.5453 29192301

[B107] Roth S. Dreixler J. C. Mathew B. Balyasnikova I. Mann J. R. Boddapati V.. (2016). Hypoxic-preconditioned bone marrow stem cell medium significantly improves outcome after retinal ischemia in rats. Invest. Ophthalmol. Vis. Sci. 57, 3522–3532. 10.1167/iovs.15-17381 27367588 PMC4961056

[B108] Rustom A. Saffrich R. Markovic I. Walther P. Gerdes H. H. (2004). Nanotubular highways for intercellular organelle transport. Science 303, 1007–1010. 10.1126/science.1093133 14963329

[B109] Ryan J. M. Barry F. P. Murphy J. M. Mahon B. P. (2005). Mesenchymal stem cells avoid allogeneic rejection. J. Inflamm. 2:8. 10.1186/1476-9255-2-8 16045800 PMC1215510

[B110] Sabapathy V. Kumar S. (2016). hiPSC-derived iMSCs: NextGen MSCs as an advanced therapeutically active cell resource for regenerative medicine. J. Cell. Mol. Med. 20, 1571–1588. 10.1111/jcmm.12839 27097531 PMC4956943

[B111] Scholl H. P. Strauss R. W. Singh M. S. Dalkara D. Roska B. Picaud S.. (2016). Emerging therapies for inherited retinal degeneration. Sci. Transl. Med. 8:368rv366. 10.1126/scitranslmed.aaf2838 27928030

[B112] Shen Y. (2020). Stem cell therapies for retinal diseases: from bench to bedside. J. Mol. Med. 98, 1347–1368. 10.1007/s00109-020-01960-5 32794020

[B113] Shi Y. Wang Y. Li Q. Liu K. Hou J. Shao C.. (2018). Immunoregulatory mechanisms of mesenchymal stem and stromal cells in inflammatory diseases. Nat. Rev. Nephrol. 14, 493–507. 10.1038/s41581-018-0023-5 29895977

[B114] Shirley Ding S. L. Kumar S. Ali Khan M. S. Ling Mok P. (2018). Human mesenchymal stem cells expressing erythropoietin enhance survivability of retinal neurons against oxidative stress: an *in vitro* study. Front. Cell. Neurosci. 12:190. 10.3389/fncel.2018.00190 30108483 PMC6079241

[B115] Singh M. S. Park S. S. Albini T. A. Canto-Soler M. V. Klassen H. Maclaren R. E.. (2020). Retinal stem cell transplantation: balancing safety and potential. Prog. Retin. Eye Res. 75:100779. 10.1016/j.preteyeres.2019.100779 31494256 PMC7056514

[B116] Slamecka J. Salimova L. Mcclellan S. Van Kelle M. Kehl D. Laurini J.. (2016). Non-integrating episomal plasmid-based reprogramming of human amniotic fluid stem cells into induced pluripotent stem cells in chemically defined conditions. Cell Cycle 15, 234–249. 10.1080/15384101.2015.1121332 26654216 PMC4825845

[B117] Spees J. L. Lee R. H. Gregory C. A. (2016). Mechanisms of mesenchymal stem/stromal cell function. Stem Cell Res. Ther. 7:125. 10.1186/s13287-016-0363-7 27581859 PMC5007684

[B118] Spees J. L. Olson S. D. Whitney M. J. Prockop D. J. (2006). Mitochondrial transfer between cells can rescue aerobic respiration. Proc. Natl. Acad. Sci. U.S.A. 103, 1283–1288. 10.1073/pnas.0510511103 16432190 PMC1345715

[B119] Takagi S. Mandai M. Gocho K. Hirami Y. Yamamoto M. Fujihara M.. (2019). Evaluation of transplanted autologous induced pluripotent stem cell-derived retinal pigment epithelium in exudative age-related macular degeneration. Ophthalmol. Retina 3, 850–859. 10.1016/j.oret.2019.04.021 31248784

[B120] Tassoni A. Gutteridge A. Barber A. C. Osborne A. Martin K. R. (2015). Molecular mechanisms mediating retinal reactive gliosis following bone marrow mesenchymal stem cell transplantation. Stem Cells 33, 3006–3016. 10.1002/stem.2095 26175331 PMC4832383

[B121] Teo H. Ghosh S. Luesch H. Ghosh A. Wong E. T. Malik N.. (2010). Telomere-independent Rap1 is an IKK adaptor and regulates NF-kappaB-dependent gene expression. Nat. Cell Biol. 12, 758–767. 10.1038/ncb2080 20622870

[B122] Trivedi A. Miyazawa B. Gibb S. Valanoski K. Vivona L. Lin M.. (2019). Bone marrow donor selection and characterization of MSCs is critical for pre-clinical and clinical cell dose production. J. Transl. Med. 17:128. 10.1186/s12967-019-1877-4 30995929 PMC6469059

[B123] Turner L. (2018). The US direct-to-consumer marketplace for autologous stem cell interventions. Perspect. Biol. Med. 61, 7–24. 10.1353/pbm.2018.0024 29805145

[B124] Tzameret A. Sher I. Belkin M. Treves A. J. Meir A. Nagler A.. (2014). Transplantation of human bone marrow mesenchymal stem cells as a thin subretinal layer ameliorates retinal degeneration in a rat model of retinal dystrophy. Exp. Eye Res. 118, 135–144. 10.1016/j.exer.2013.10.023 24239509

[B125] Veleri S. Lazar C. H. Chang B. Sieving P. A. Banin E. Swaroop A. (2015). Biology and therapy of inherited retinal degenerative disease: insights from mouse models. Dis. Model. Mech. 8, 109–129. 10.1242/dmm.017913 25650393 PMC4314777

[B126] Vignais M. L. Caicedo A. Brondello J. M. Jorgensen C. (2017). Cell Connections by tunneling nanotubes: effects of mitochondrial trafficking on target cell metabolism, homeostasis, and response to therapy. Stem Cells Int. 2017:6917941. 10.1155/2017/6917941 28659978 PMC5474251

[B127] Von Bahr L. Batsis I. Moll G. Hägg M. Szakos A. Sundberg B.. (2012). Analysis of tissues following mesenchymal stromal cell therapy in humans indicates limited long-term engraftment and no ectopic tissue formation. Stem Cells. 30, 1575–1578. 10.1002/stem.1118 22553154

[B128] Wu Z. Chen G. Zhang J. Hua Y. Li J. Liu B.. (2017). Treatment of myocardial infarction with gene-modified mesenchymal stem cells in a small molecular hydrogel. Sci. Rep. 7:15826. 10.1038/s41598-017-15870-z 29158523 PMC5696474

[B129] Xian B. Huang B. (2015). The immune response of stem cells in subretinal transplantation. Stem Cell Res. Ther. 6:161. 10.1186/s13287-015-0167-1 26364954 PMC4568575

[B130] Xu C. Yu P. Han X. Du L. Gan J. Wang Y.. (2014). TGF-beta promotes immune responses in the presence of mesenchymal stem cells. J. Immunol. 192, 103–109. 10.4049/jimmunol.1302164 24293629

[B131] Yu B. Shao H. Su C. Jiang Y. Chen X. Bai L.. (2016). Exosomes derived from MSCs ameliorate retinal laser injury partially by inhibition of MCP-1. Sci. Rep. 6:34562. 10.1038/srep34562 27686625 PMC5043341

[B132] Zhang J. Chan Y. C. Ho J. C. Siu C. W. Lian Q. Tse H. F. (2012). Regulation of cell proliferation of human induced pluripotent stem cell-derived mesenchymal stem cells via ether-a-go-go 1 (hEAG1) potassium channel. Am. J. Physiol. Cell Physiol 303, C115–125. 10.1152/ajpcell.00326.2011 22357737

[B133] Zhang Y. Chiu S. Liang X. Gao F. Zhang Z. Liao S.. (2015). Rap1-mediated nuclear factor-kappaB (NF-kappaB) activity regulates the paracrine capacity of mesenchymal stem cells in heart repair following infarction. Cell Death Discov. 1:15007. 10.1038/cddiscovery.2015.7 27551443 PMC4981000

